# Development of a digital hearing aid to meet the Brazilian Government's Ordinance 587 (APAC) – Health Department

**DOI:** 10.1590/S1808-86942010000300011

**Published:** 2015-10-20

**Authors:** Silvio Pires Penteado, Ricardo Ferreira Bento

**Affiliations:** 1 Electronic engineer. Doctoral student of otorhinolaryngology at the Medical School of the São Paulo University; 2 Full professor of otorhinolaryngology at the Medical School of the Sao Paulo University. Head of the otorhinolaryngology and ophthalmology department of the Clinical Hospital, Medical School of the Sao Paulo University

**Keywords:** hearing loss, low cost technology, prostheses and implants, public policies

## Abstract

The treatment of sensorineural hearing loss is based on hearing aids, also known as individual sound amplification devices. The hearing aids purchased by the Brazilian Government, aiming at fulfilling public policies, are based on dedicated components, which bring about benefits, but also render them expensive and may impair repair services after manufacture's warranty expires.

**Aim:** to design digital behind-the-ear hearing aids built from standardized components coming from the very supply chain of these manufacturers.

**Study design:** experimental.

**Materials and Methods:** to identify the supply chain of these manufacturers, request samples and set up hearing aids in the laboratory.

**Results:** The developed hearing aids did not show lesser electroacoustic characteristics when compared to those acquired by the Government, also being tested by the same reference international technical standard.

**Conclusion:** It is possible to develop digital behind-the-ear hearing aids based on off-the-shelf components from hearing aid manufacturers' supply chain. Their advantages include low operational costs – for acquisition (with clear advantages for the Government) and service (advantage for the patient).

## INTRODUCTION

Although predictable, presbycusis is one of the hardest sensorineural dysfunctions to accept^1^ and one of the three most common chronic conditions in the elderly, together with arthritis and arterial hypertension.^2^ According to the degree, duration and associated etiology, hearing loss in children may result in disorders that invariably result in poor school performance; this becomes an additional challenge for educators and family members, even if these losses are moderate and unilateral.^3^

Biological recovery of hearing is still being researched; thus, hearing prostheses – known as hearing aids or individual sound amplification devices (ISADs) – are the main tools for sensorineural auditory rehabilitation or habilitation. Although the electronics and components of these devices are relatively simple, ISADs are the object of strong complaints about the end price for patients.^4^, ^5^, ^6^

A study^7^ showed that the most important single factor that reduces the satisfaction with using ISADs is its acquisition cost. Other researchers8 have stated that “the price of hearing aids is unacceptable (…) and is the most important barrier against its use.” Kochkin^9^ concluded that the relative importance that hearing impaired patients give to acquisition and maintenance costs is singly higher than the combined factors of motivation, stigma about the use of hearing aids, and the perception of being a hearing impaired person.

A survey of 214 participants by Boymans et al.^10^ showed that 93% of these patients prefer binaural fitting. Erdman e Sedge^11^ found a similar number (90%), which confirms that binaural fitting yields better results compared to unilateral fitting. However, acquisition of two ISADs is a significant cost for most retail patients.^12^

A report by The British Society of Hearing Aids Audiologists^13^ underlines several methods that some European governments use to donate ISADs. In Denmark, for instance, 90% of all ISADs are adapted in public hospitals; patients that prefer private clinics enjoy a? 700 subsidy for each ISAD. In Switzerland, where government subsidy may reach? 1,960 per ISAD, there are three levels of ISAD technology (simple, complex, and very complex), defined based on a scoring system according to the social and economic effects of hearing loss. For instance, a waitress scores higher than a sedentary elderly patient. Germany's policy is to apply the criterion of rehabilitating hearing impaired patients in a “necessary and adequate” manner with subsidies ranging from ? 470 to ? 530 per ISAD. In Sweden, the public sector provides subsidies ranging from ? 200 to ? 400 per ISAD, while in England the government donates behind-the-ear (BTE) or retroauricular ISADs with the batteries, ear molds, and repair services. A 1982 report found that half the hearing impaired population in Australia were eligible to receive government donations as ISADs, as well as batteries and technical support.^14^ There are no government ISAD donations in the United States; proceedings are taking place in Congress since May 2007, namely the “Hearing Aid Assistance Tax Credit Act -HR2329/S.1410,” the purpose of which is to offer a U$ 1,000 credit for acquiring two ISAD.

Batista and Sampaio^15^ stated that the Ministry of Health proposed an ISAD-donation policy to patients of the Unified Health System (SUS) as of the

Administrative Rule n° 3.764 of October 1998. In 2002, the Ministry of Health invested R$ 47,081,886.75 in a program that provided 35,297 hearing aids at 84 Service Providing Units (UPS). In 2003, expensed with hearing health reached R$ 103,371,561.75, and in 2004 these costs were R$ 162,705,737.00. These numbers do not include ISAD fitting services: the diagnosis, selection and indication of hearing aids, monitoring and speech therapy. In 2004, about 10% of the expenditure with hearing health focused on these services; the remaining amount was for purchasing ISADs.

We highlight the Administrative Rules no. 587 and no. 589 (both from October 2004), which made it possible to implement and operate state networks at a countrywide level. Administrative Rule no. 587 became known as the AHCP (Authorization for High Complexity Procedures). Somewhat similarly to what occurs in Switzerland, the Administrative Rule no. 587 classifies ISADs in three categories: Technology A, Technology B, and Technology C; the first offers basic features (for instance, non-programmable and single channel), the third provides advanced features (for instance, noise-reduction and acoustic feedback algorithms), and the second offers intermediate features. This same Administrative Rule states that the prescription percentages would be 50% for Technology A, 35% for Technology B, and 15% for Technology C.

An ISAD is a portable individual sound amplification system based on three electronic components – a microphone, a digital signal processor (DSP), and a receptor.16 The other parts of an ISAD are low added-value electric, plastic, or silicone-based components. Electronic components – those with a higher value – are not produced by the ISAD manufacturers themselves, but by their supply chains.17 Certain DSPs are made for a manufacturer for a specific design,^18^ which is referred to as a dedicated integrated circuit (or “application-specific integrated circuit”); other DSPs are standard or generic, and may be customized for any project of interest. Generally speaking, all transducers are standard components.

The WHO proposed using standard components^19^ when it defined a set of guidelines to expand ISAD use at reasonable prices in developing countries. At that point, it was suggested that maintenance be carried out by manufacturer's decentralized shops.

The advantages of designing products with more components are economy of scale and scope, with occur when major negotiations result in decreased acquisition and production costs;^20^,^21^ this in turn may result in lower acquisition costs for governments or retail customers.

The price policy of government-donated ISADs is proportional to the level of technology: Technology A – R$ 525.00, Technology B – R$ 700.00, and Technology C – R$ 1,100.00, according to the Administrative Rule no. 308 (2007). Some Technology C ISADs may cost more than R$ 5,000.00 for retail customers; in these cases, repair costs our of warranty periods may be excessive for patients who received these devices as donations, since these patients will face the same price policy applied to other retail customers. In this sense, using standard components may assure reasonable customer service prices after the warranty period is over, since patients become end owners interested in maintaining their products.

The life cycle of an ISAD is short, ranging from 3 to 5 years;^22^,^23^ thus, high acquisition and maintenance costs may indicate inefficient use of public or private healthcare funds.

The theme of this article is justified by the possibility of developing digital ISADs from standard industrial components that may become a reference for the Federal Government when elaborating public policies for hearing health. The main advantage is low acquisition and maintenance operating costs. The supply chain of international ISAD manufacturers will be approached to request samples of standard components to develop a digital BTE ISAD.

The purpose of this study was to develop BTE ISADs using standard components that meet the specifications of the Federal Government, according to the Administrative Rule no. 587.

## MATERIAL AND METHOD

The institutional review board of the Research Project Analysis Department (CAPPesq) approved this study (protocol no. 1044/66), although no tests were carried out on human beings. Outsourced technical services were used to assemble and for quality control certification (regulation IEC60.118-7). The choice of design for the BTE device makes it possible to compare it with other similar BTE devices made by international manufacturers, and do not require physical changes to fit on patients; all that is required is the ear mold to begin ISAD electro-acoustic adjustments. Regulation IEC 60.118-7 is one of the most used in ISAD manufacturing for certification.

Having defined the ISAD design as a BTE device, we went on to identify the components for its development. These were divided into three groups:

1. Electronic components: DSPs and transducers (microphone and receptor);
2. Electric components: programming socket, volume control, programming button, telecoil;
3. Other components: BTE case, presentation box, and consumables (for instance, transducer suspension and silicon tubes).

Electronic components are the most important and have the highest added value. DSPs from the following manufacturers were used: Texas Instruments Inc., (Houston, Texas, USA), Etymotic Research Inc., (Elk Grove Village, Illinois, EUA), and Gennum Corp. (Burlington, Ontario, Canada). Transducers were provided by the companies Knowles Electronics LLC (Itasca, Illinois, EUA), Sonion A/S (Roskilde, Denmark), and Tibbetts Industries Inc. (Camden, Maine, EUA). All electrical components were provided by Deltek, a division of Knowles. The BTE box was provided by Int'Tech Industries Inc. (Ramsey, Minnesota, EUA), and the presentation box and consumables were purchased locally.

This project was developed based on the specification set forth in the Administrative Rule no. 587.

Table 1 shows the applications (dedicated programs for product development).

**Table 1:** List of the applications used in this development.

Description	Version
ARK online®	4.8.3
ARK Component Manager	Not informed
Controller Toolbox	1.0.6
Interactive Data Sheet	4.2.0
SOUNDFIT®	4.0.0.14
SOUNDFIT® Customization Tool	4.0.0.7

**Figure 1:**
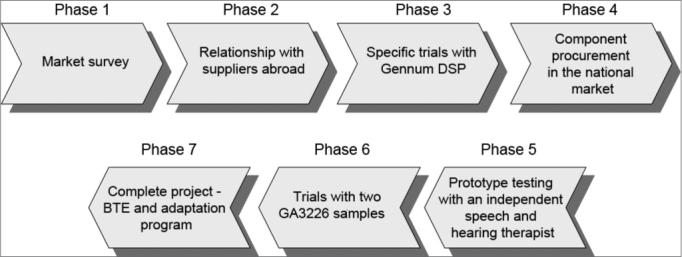
BTE Development Flow.

BTE development was done in seven phases (Fig. 1).

Phase 1 – Market research – the features of the Brazilian market were surveyed (ANVISA, customs laws, end price with added tax, understanding the retail distribution network, estimating the potential retail market in Brazil, understanding the currently sold products in the domestic market, understanding international ISAD certification technical standards, product specifications).

Phase 2 – Relationship with suppliers abroad – samples of ISAD components were asked for and received, which enabled us to assemble and run several tests on functioning structures. This phase was important to learn about products in the ISAD manufacturer supply chain, to see whether our project requirements could be met.

Phase 3 – Specific trials with the Gennum DSP – we chose to use the Gennum GA3216 DSP, as it was closer to our project specifications: it has a standardized DSP (off-the-shelf or generic) that was flexible enough to met our project specifications; it has a set of low-cost development tools – reasonable enough for our development costs – and a easy-to-use fitting software that could be translated into Portuguese.

Although international manufacturers sent samples, we had to purchase other imported components in the Brazilian market to run several laboratory trials and test as many component combinations as we could. Transducers were the most frequently tested components. After positive results were attained, we started to write the BTE device Service Manual to record assembly procedures, components, quality control criteria, and others. This was Phase 4 (acquisition of components in the Brazilian market).

We informally asked Maria Helena Untura Caetano, PhD, a speech therapist, to test the BTE device and to comment on our product, which was essential at this phase of development. This was Phase 5 (having an independent speech therapist test a prototype).

Phase 6 (trials with two GA3226 samples) – we started several trials with a recently launched DSP (by Sound Design, which acquired Gennum in 2007), the GA3226, which was kindly provided to us for testing. Two operating prototypes were assembled, with positive results, which encouraged us to use it as the final DSP in our project.

Phase 7 (complete project – BTE device and fitting program) – we assembled, tested and certified two ISAD, according to the international standard IEC60.118-7. We also finalized the fitting procedure and the Service Manual.

One of the reasons we chose the Sound Design DSP was that it contains dedicated applications for ongoing product development, not requiring investments in peripheral devices; contacting its server in the development phase is relatively simple and low-cost. These applications are Sound Design's Application Resource Kit (ARK). A DSP needs to be customized for each project; it is provided in such a way that it will not operate predictably when connected electrically to other components. The ARK is used to generate an ordered task flow that yields two main events: firmware recording of the features defined in the project, and their recognition and programming when fitting.

The ARK-based BTE device development steps were as follows:

### Step 1. Logging in to the Sound Design server

A personal computer is used to log in to the Sound Design server. The ARK starts a tutorial for the next steps. A specific application – the ARK online – was used in these first three steps.

### Step 2. Defining the map

This step defined the electroacoustic features of the ISAD, such as the limits of the crossover filters, compression rate values, and frequency equalizer step values. We were connected to the Sound Design server during this and the next two steps.

### Step 3. Defining the library

**Figure 2:**
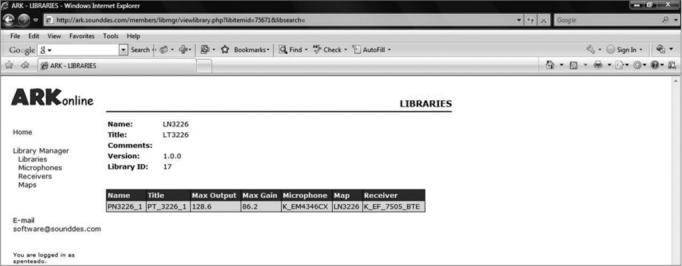
Screen with the library defined for BTE.

At this point we added the features defined in the map to the transducers defined in the project. Figure 2 shows the library screen defined for this project; the expected gain and output based on this configuration are shown.

### Step 4. Downloading the library to the personal computer (PC)

We downloaded this library from the Sound Design server to a specific directory in the PC: C:\\Windows\ ARK. The library comprises two files: a dynamic library (.dll) and a second file with the features (.src). We used the ARK Component Manager application for this step.

### Step 5. Welding the components

Up to this step the project specifications were defined in the map and the library, which were in the PC. The electrical and electronic components were assembled according to the manufacturer's instructions.

### Step 6. Checking connectivity

**Figure 3:**
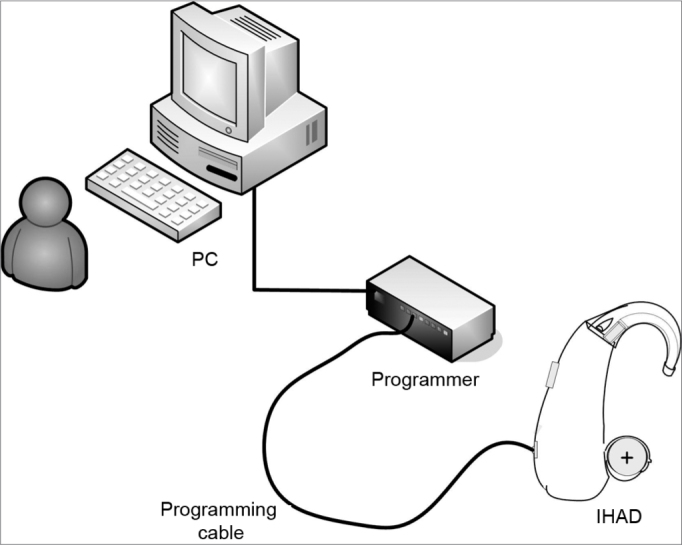
Connection between PC and BTE.

The Controller Toolbox application checks whether the PC and the GA3226 are electrically connected. This step is important, as it avoids continuing to assemble an ISAD with non-operating or failed electrical and electronic components. There is an ISAD programmer between the PC and the electrical/electronic circuit; for this project, we defined a standard programmer (HI-PRO, GN ReSound A/S, Taastrup, Denmark). There is a programming cable (a standard Knowles CS44 cable). Figure 3 shows a simplified image of the connection between the PC and the BTE device. The steps shown on the screen in this application were followed to check connectivity. This and the next step do not require a connection to the Sound Design server.

### Step 7. Customizing other ISAD features

At this point a few features still have to be defined before executing the GA3226 firmware recording process: the type of button for changing the comfort program (temporary or continuous contact), the indicator for changing sound frequencies and intensities in the comfort program (beep), the initial compression rate in each channel, and others. We used the Interactive Data Sheet program to customize these features, as seen in the schematics shown on Figure 3. In this step we recorded all the project-defined specifications on the GA3226 firmware.

### Step 8. Obtaining the Sound Design fitting software

We downloaded the standard Soundfit software from the server to the PC. At this point we were connected to the Sound Design server.

### Step 9. Customizing the fitting software

Soundfit was in English and had to be translated into Portuguese to meet Brazilian legal requirements. Figures were inserted, some screen colors were changed, and other esthetic changes were made to increase user-friendliness. The Soundfit Customization Tool was used to define fitting in Portuguese: it was named AdaptEASY. This step does not require a connection to the server.

### Step 10. Finalizing the ISAD-assembly process

Once the GA3226 specifications and connectivity to the AdaptEASY were defined and tested, we finalized the assembly of the BTE device by inserting the electrical and electronic components (hereafter named the electronic architecture) within the box. We were careful not to kink the cables and to make it easy to open and access the BTE device for repair purposes.

### Step 11. Testing the BTE device with a stethoscope

Tests with a stethoscope were done to indentify subjective qualities (volume, sound quality, and sound feedback), and to support the failure identification process, which may imply in reassembling or exchanging parts. In this phase, we found that the volume control responded slowly, only after a specific angular position; we then replaced the value of this component from 200 k? to 100 k?. After this change, the volume control worked perceptibly throughout its range.

### Step 12. Connecting the BTE device to the customized fitting program

At this point we executed the connection tests between the AdaptEASY and the BTE device; this is similar to what speech therapists face in their clinical routine. Hypothetical fittings to patients were done intensively, and no software or hardware issues were found.

### Step 13. Certifying the ISAD under international standards

Quality control (QC) tests were done using acoustic testing equipment (technical reference no. IEC60.118-7). Current regulations require keeping a printed report of the recorded parameters, their dynamic curves, and the technical standards that were applied for tracking and future repair services. The technical specifications of the BTE device and the acceptable tolerances are available in the Service Manual.

## RESULTS

**Figure 4:**
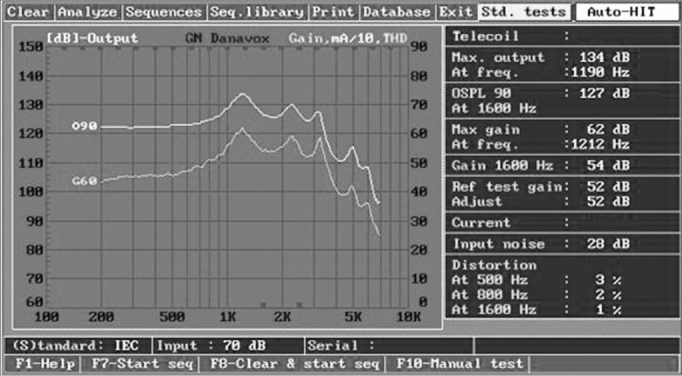
Characteristics curves of the developed BTE. IEC60.118-7 Standard

The curves show (Fig. 4) that the frequency response of our BTE device ranges from 250 to 7,500 Hz, the stable gain is 62 dB, the output is 134 dB, and harmonic distortion is low (below 4% in the worst case); it therefore is applicable up to moderate-severe hearing loss, according to the reference classification.^24^

As it consumes little current, the autonomy of the BTE device is 440-hours with a size 675 battery. It has common features with the most recent ISADs in the market: adaptive sound feedback manger, 12-band graphic equalizer, four comfort programs, one, two or four-channel WDRC signal processing strategy (dynamic compression), AGC-O control, sound indicator (beep) when changing the comfort program and to indicate low battery power. Table 2 shows the specifications of the BTE device we developed.

**Table 2:** Specifications of the developed BTE

Parameter	Values
Maximum output (dB)	134
Maximum gain (dB)	62
Battery drain (mA)	1.1
Battery size	675
Battery duration (hours)	440
Background noise (dB)	28
Number of comfort programs	4
Number of channels	4
Feedback noise manager	Present
Graph equalizer	12 bands

IEC60.118-7 Standard

The end cost of our BTE device was U$ 140.13, including acquisition of extra components and outsourced assembly services.

## DISCUSSION

**Figure 5:**
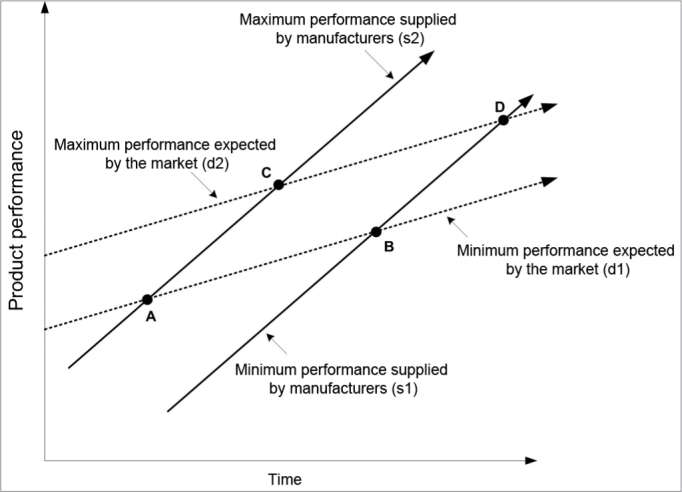
The ABCD specifications from the Ministry of Health.

Christensen^25^ concluded that industrial organizations seek higher and higher profits by offering consumer increasingly sophisticated products – line segments (s1) and (s2) – in which the density of technology progresses faster than market demand – line segments (d1) and (d2) on the model in Figure 5. As the Administrative Rule no. 587 does not take into account products with inferior technology and at the same time limits the set of superior technologies, we can see that ISADs with minimal specifications are located in the ABCD polygon.

In this context, Shih^26^ states that technological innovations placed in the market (“technology-push”) vary in degree and intensity compared to market-driven innovation (“demand-pull”), and that both accurately define the whole life cycle of technological innovation in society.

According to Romero,^27^ generic drugs were proposed as a strategy to rationally increase the consumption of medication, thereby regulating prices. A Brazilian study^28^ in 2003 underlined the positive impact of such government policies, which “are changing the industrial structure of that sector” wherein generic drugs have become an important factor for providing popular access to medication and have helped reduce public expenditure. By analogy, ISADs built using a common (or generic) electric and electronic platform may help reduce public expenses and affect retail market prices.

Because our BTE device is a single model that meet fully the specifications of Technologies A and B in the Administrative Rule no. 587, and 85% of the specifications in the Administrative Rule no. 587. It can meet all of these specifications if an extra microphone (as part of adaptive directionality) and the expansion feature are inserted, which does not require changing any other component, since the case is made to receive a second microphone; all that needs to be one is to change the library and the fitting program.

Thus, it is possible to design ISADs according to government-required specifications. BTE devices may be developed for hearing loss below the limits defined in this project; all that is required is to change the maps and libraries while retaining the electric and electronic library. Furthermore, the same electrical and electronic configuration may be used for severe hearing loss, as the GA3226 DSP supports stable gains up to 84 dB,^29^ as confirmed in the library for this project (Fig. 2). Again, the same electrical and electronic configuration may be use to develop in-the-canal (ITC or intracanal) and in-the-ear (ITE or auricular) ISADs, just by altering the maps and libraries. The receptor needs to be replaced when designing completely-in-the-canal (CIC or microcanal) ISADs, because of its small size.

ISADs are tax-free (for the IPI, ICM, and part of the ISS taxes). A Web consultation in the Ministry of Development, Industry and Foreign Trade30 showed that the mean values declared by importers were US$ 166.38 (2005), US$ 177.96 (2006), US$ 159.35 (2007), and US$ 152.14 (2008), all of which are higher than the end value of our BTE device (US$ 140.13).

Audiologically speaking, the AdapEASY fitting program consists of three steps only: gathering patient information and an audiogram, selecting the ISAD, and adjusting the program. Fitting may be installed in a modestly configured PC: Windows XPTM (or VistaTM) operating system, a 4 GB hard drive, 1 GB RAM, and a standard cable and programmer; the advantages are low operating costs and easy updating.

The Administrative Rule no. 587 should not be mistaken with the Law no. 8,666; the latter sets forward general guidelines about tenders and administrative contracts based on the lowest price.

Having produced two units, we went on to produce 25 BTE devices, which are currently being tested on human beings; no results have been published to date.

## CONCLUSION

We were able to develop a BTE digital device from standard components in the supply chain of international manufacturers of ISADs. Our BTE device meets the Technology A and B specifications, which is about 85% of such devices sold to the Brazilian government by international manufacturers. It was also IEC 60.118-7-certified.
